# Impact of Alternative Splicing Variants on Liver Cancer Biology

**DOI:** 10.3390/cancers14010018

**Published:** 2021-12-21

**Authors:** Jose J. G. Marin, Maria Reviejo, Meraris Soto, Elisa Lozano, Maitane Asensio, Sara Ortiz-Rivero, Carmen Berasain, Matias A. Avila, Elisa Herraez

**Affiliations:** 1 Experimental Hepatology and Drug Targeting (HEVEPHARM), Institute for Biomedical Research of Salamanca (IBSAL), University of Salamanca, 37007 Salamanca, Spain; maredi@usal.es (M.R.); merarissoto@gmail.com (M.S.); elisa_biologia@usal.es (E.L.); masensio002@usal.es (M.A.); saraortizriv@usal.es (S.O.-R.); elisah@usal.es (E.H.); 2 Center for the Study of Liver and Gastrointestinal Diseases (CIBERehd), Carlos III National Institute of Health, 28029 Madrid, Spain; cberasain@unav.es (C.B.); maavila@unav.es (M.A.A.); 3 Instituto de Investigaciones Sanitarias de Navarra IdiSNA, 31008 Pamplona, Spain; 4 Hepatology Program, CIMA, University of Navarra, 31008 Pamplona, Spain

**Keywords:** alternative splicing, carcinogenesis, chemotherapy, cholangiocarcinoma, hepatocellular carcinoma, metastasis, multidrug resistance

## Abstract

**Simple Summary:**

Among the top ten deadly solid tumors are the two most frequent liver cancers, hepatocellular carcinoma, and intrahepatic cholangiocarcinoma, whose development and malignancy are favored by multifactorial conditions, which include aberrant maturation of pre-mRNA due to abnormalities in either the machinery involved in the splicing, i.e., the spliceosome and associated factors, or the nucleotide sequences of essential sites for the exon recognition process. As a consequence of cancer-associated aberrant splicing in hepatocytes- and cholangiocytes-derived cancer cells, abnormal proteins are synthesized. They contribute to the dysregulated proliferation and eventually transformation of these cells to phenotypes with enhanced invasiveness, migration, and multidrug resistance, which contributes to the poor prognosis that characterizes these liver cancers.

**Abstract:**

The two most frequent primary cancers affecting the liver, whose incidence is growing worldwide, are hepatocellular carcinoma (HCC) and intrahepatic cholangiocarcinoma (iCCA), which are among the five most lethal solid tumors with meager 5-year survival rates. The common difficulty in most cases to reach an early diagnosis, the aggressive invasiveness of both tumors, and the lack of favorable response to pharmacotherapy, either classical chemotherapy or modern targeted therapy, account for the poor outcome of these patients. Alternative splicing (AS) during pre-mRNA maturation results in changes that might affect proteins involved in different aspects of cancer biology, such as cell cycle dysregulation, cytoskeleton disorganization, migration, and adhesion, which favors carcinogenesis, tumor promotion, and progression, allowing cancer cells to escape from pharmacological treatments. Reasons accounting for cancer-associated aberrant splicing include mutations that create or disrupt splicing sites or splicing enhancers or silencers, abnormal expression of splicing factors, and impaired signaling pathways affecting the activity of the splicing machinery. Here we have reviewed the available information regarding the impact of AS on liver carcinogenesis and the development of malignant characteristics of HCC and iCCA, whose understanding is required to develop novel therapeutical approaches aimed at manipulating the phenotype of cancer cells.

## 1. Introduction

According to cancer incidence and mortality statistics worldwide (both genders, all ages) registered by Globocan (https://gco.iarc.fr/ accessed on 18 September 2021), in 2020, liver cancer was ranked sixth in incidence with 905,677 new cases (4.7% of new cases) but third regarding mortality with 830,180 deaths (8.3% of all cancer-related deaths). Liver cancer includes several forms of cancers, but in most adult patients, these tumors are either hepatocellular carcinoma (HCC) (85–90%), derived from hepatocytes, or intrahepatic cholangiocarcinoma (iCCA) (10–15%), derived from cholangiocytes.

Because aberrant splicing generates mRNA isoforms involved in liver cancer development and malignancy [1], several studies have attempted to establish a relationship between alternative splicing (AS) signatures and the prognosis of HCC [2,3,4,5] and iCCA [6,7]. Editing immature mRNA (pre-mRNA) by splicing is carried out by the spliceosome, a multi-megadalton ribonucleoprotein complex in charge of introns removal and exons ligation as part of an integrated mechanism involved in gene regulatory network [8]. In “constitutive splicing”, this occurs at canonical sites of pre-mRNA, whereas in AS the cut-and-paste process involves different splicing sites, which result in diverse forms of partial or complete removal or retention of exons and introns [9]. The relevance of AS is easily understood if one considers that a single pre-mRNA can produce more than one splicing variant (SV) of mature mRNA, resulting in structurally and functionally dissimilar proteins. Since approximately 97% of human genes can undergo AS [9,10] under normal physiological conditions, this diversity substantially increases the coding capacity of our genome [11]. Nevertheless, AS is also a source of noxious proteins, which have been associated with the pathogenesis of several diseases, including cancer. Moreover, re-splicing of mature mRNA in cancer cells promotes the activation of distant weak alternative splice sites, potentially generating mutation-independent diversity in cancer transcriptomes. This process might result from the impairment during carcinogenesis of a not-yet well-understood mechanism existing in normal cells to prevent potentially harmful mRNA re-splicing events [12]. Cancer-specific re-splicing, such as that of *TSG101* mRNA in nasopharyngeal carcinoma, has been proposed to be associated with enhanced invasiveness and metastasis [13].

The present review deals with the current understanding of the impact of AS on liver carcinogenesis and the development of malignant traits frequently found in HCC and iCCA.

## 2. The Intron Removal Process

The vast majority of pre-mRNA in humans are processed by the so-called “major spliceosome”, which binds to specific sequences located in the introns and plays an essential role in the exon-recognition process [14]. These are the 5′ donor site (5′SS, GU) and the 3′ acceptor site (3′SS, AG) located at the intron-exon junctions [15]. In the case of a few genes (1%) the “minor spliceosome” binds to equivalent sequences for 5′SS and 3′SS, which in this case are AU and AC, respectively [16,17,18]. Two additional signatures of the intron are the branch point site (BPS), located 18–40 nt from the end of the intron, and the polypyrimidine tract (PPT), usually 15–20 nt long, located about 5–40 nt upstream of the 3′SS [15]. The sequential interaction of spliceosome elements, such as small nuclear ribonucleoproteins (snRNP) consisting of small nuclear RNAs (U1, U2, U4, U5, and U6 snRNA) and Sm proteins, is affected by the balance between SR protein and heterogeneous nuclear ribonucleoproteins (hnRNPs), which determines the outcome of mRNA maturation. During exon-definition, the first step is the formation of the E complex, which starts with the interaction of snRNP with the 5′SS in the pre-mRNA. Then, the splicing factor 1 (Sf1) binds to the BPS, which is followed by the recruitment of the U2 heterodimer (U2AF), whose 35 kDa (U2af1) and 65 kDa (U2af2) subunits bind to the 3’SS and PPT, respectively [19]. The next step is the formation of the A complex, in which U2 snRNP displaces Sf1 in an ATP-dependent manner. The RNA helicases PRP5/DDX46 and DDX39B/UAP56 promote the interaction of U1 snRNP with U2 snRNP [20] and stabilize the interaction between U2 snRNP and pre-mRNA BPS [21], respectively. The recruitment of U4/U6-U5 tri-snRNP generates the pre-catalytic B complex (B1), from which U1 and U4 snRNPs are released, resulting in the activated B complex (B2) [22]. The subsequent step leads to the first reaction of splicing, producing the C complex together with an intermediate pre-mRNA with lariat structure [23]. Another trans-esterification reaction catalyzed by the C complex releases the intron lariat structure, which is rapidly degraded by the cell [24,25,26]. The pre-mRNA processing factors 3, 4, 6, 8, and 31 (PRPF3, PRPF4, PRPF6, PRPF8, PRPF31) participate in the splicing of the pre-mRNA as components of the U4/U6-U5 tri-snRNP complex and the spliceosome B complexes [27].

Besides the difference in guanine and cytosine content between exons and introns together with the existence of tag sequences (5′SS, 3′SS, BPS, and PPT), there are cis-regulatory sequences, which depending on their position and function, are named exonic splicing enhancers (ESE), exonic splicing silencers (ESS), intronic splicing enhancers (ISE), and intronic splicing silencers (ISS). Due to their interaction with regulatory proteins, these sites play a crucial role in defining pre-mRNA fragments, which are then removed as introns from the final mature mRNA [15,18,28,29]. Among the splicing factors involved in this process are SR proteins, characterized by a domain rich in arginine/serine dipeptides, which bind to ESE and ISE, favoring short alternatives of splicing [30]. In contrast, hnRNPs bind to ESS and ISS, inhibiting short options of splicing and hence favoring the formation of long SVs [31].

Besides, several kinases and phosphatases, by modulating the phosphorylation and dephosphorylation of SR proteins and other spliceosome components, determine their activity and cellular localization and hence participate indirectly in regulating the spliceosome [32].

## 3. Liver Cancer-Associated Alterations in the Spliceosome Machinery

During carcinogenesis, a common feature is the need for a high rate of pre-mRNA processing in combination with the frequent appearance of alterations in spliceosome elements, which altogether account for the generation of a large variety of SVs (more than 15,000) associated with different cancer types [33,34]. Although some of these SVs are noxious for the cells harboring them, in most cases, these variants have a positive impact on tumor biology, enhancing their malignant characteristics [35,36,37]. In the case of liver cancer, the global profile of alternative RNA splicing events indicates the existence of differential AS in HCC and iCCA compared to adjacent non-tumor liver tissue, affecting several hundred genes. This information has been proposed as a valuable tool to distinguish different types of liver cancer [2,38].

Cancer-associated changes in the spliceosome can be due to: (i) altered expression of splicing factors [39]; (ii) mutations affecting genes coding spliceosome components and associated regulatory proteins; (iii) disruption of either SS or splicing regulatory sites (enhancers or silencers) [40,41]; and (iv) impairment of signaling pathways involved in the splicing machinery regulation [40,42]. In this regard, marked alterations in the expression pattern of spliceosome factors, in particular those proteins accounting for exon recognition, have been described both in HCC and iCCA, where they may affect patient outcomes (Table 1) [43,44]. Moreover, transcriptome-wide analysis has revealed the presence in HCC of a high number of aberrant AS events, which has been associated with impaired expression of splicing factors [3].

Besides alterations in their expression levels, mutations in RNA-binding proteins (RBPs) are commonly found in cancer, which contributes to tumor initiation and progression [45]. More precisely, 286 RBP-encoding genes have been found differentially expressed (231 up-regulated and 55 down-regulated) in HCC samples versus non-tumor tissue [3]. These alterations in RBP abundance presumably lead to AS dysregulation.

A meta-analysis has shown that many genes of the spliceosome machinery, such as SF3B proteins, which are essential components of the U2 complex, are up-regulated in HCC [46]. One of these proteins (SF3B1) is particularly relevant in the context of this review because it has been identified as a driver in many cancers [47]. SF3B1 plays a critical role in exon recognition by favoring U2 snRNA binding to the BPS [48] and by allowing the identification of short exons flanked by long introns with differential GC content through interaction with nucleosomes [49]. SF3B1 up-regulation in HCC has been associated with higher tumor aggressiveness and shorter overall survival (OS). This may be due in part to the favored generation of some oncogenic SVs of specific proteins, such as KLF6-SV1 [47]. Recent results obtained in human gastric cancer cells (AGS and MKN28) have revealed that inhibition of SF3B1 reduced their proliferation rate by inducing apoptosis and G2/M phase arrest through altering homeobox A10 (HOXA10) mRNA splicing. Thus, SF3B1 knockdown down-regulated HOXA10 mRNA expression whereas enhanced the expression of long noncoding RNA (lncRNA) isoforms of HOXA10 (exons 1 and 3) and HOXA10 (exons 2 and 3). Moreover, SF3B1 inhibition increased PTEN levels and reduced AKT protein phosphorylation [50].

The pharmacological inhibition of SF3B1, using pladienolide-B, results in decreased cell proliferation, migration, and colony-formation ability in HCC cells in vitro and impaired tumor growth in vivo [47]. These findings support the role of SF3B1 in liver carcinogenesis. They suggest that SF3B1 is a promising novel druggable target as well as a prognostic biomarker in HCC [51], as has been reported in other malignancies, such as pancreas [52], breast [53], and prostate [54] cancers.

Interestingly, not only changes in SF3B1 expression but also somatic mutations affecting this component of the splicing machinery play an essential role in liver cancer progression. Indeed, SF3B1 is the most frequently mutated splicing factor in many cancers [52,53,55]. These mutations have been associated with increased DNA damage and altered response to DNA damage [56]. SF3B1 mutations have also been found in HCC [57], where they are especially frequent at the late stage of carcinogenesis [58].

In addition to SF3B1, other SF3B proteins have been found associated with HCC. Thus, SF3B2 and SF3B4 are overexpressed in HCC and, similarly, in precancerous lesions in early-stage HCC [46]. Aberrant overexpression of SF3B4 inactivates Krüppel-like factor 4 (KLF4), a tumor suppressor. By inducing KLF4 aberrant splicing [59], SF3B4 may promote liver tumorigenesis, which has led to suggest its usefulness as an early-stage marker of HCC. Additionally, reduced expression of miRNA-133b triggers enhanced expression of SF3B4 in HCC, favoring metastasis, poor prognosis, and reduced OS [60,61].

Regarding core proteins involved in E complex formation, no relationship between clinical parameters of HCC and changes in SF1 and U2AF1 expression has been found [43]. In contrast, SNRPB and SNRPD1 expression is higher in HCC than in the adjacent liver tissue and correlates with shorter OS [43]. Furthermore, SNRPB up-regulation has been recently described to be mediated by c-Myc in HCC [62]. In that study, ectopic expression of SNRPB induced HCC cell proliferation and migration. In contrast, gene down-regulation promoted cell apoptosis, suggesting that SNRPB could act as an indirect oncogene in HCC [62]. It is noteworthy that SNRPB expression was found significantly increased in metastatic versus non-metastatic liver tumors, which suggests a potential role of SNRPB in HCC metastasis [62]. This is not a unique HCC characteristic because the expression of SNRPB is also increased in gliomas [63,64] and in non-small cell lung cancer (NSCLC) [65], where the tumorigenic capacity of SNRPB seems to be mediated, in part, by AS promotion leading to the formation of a RAB26 SV that retains intron 7 [65].

In both human and animal models, the step II splicing regulator SLU7 has been found down-regulated in HCC, as well as in preneoplastic conditions, such as liver cirrhosis [66,67]. The reduced SLU7 expression has been associated with the activation of the epidermal growth factor receptor (EGFR) by its ligand amphiregulin (AREG) during inflammation [66], which has also been linked to the down-regulation of other splicing regulators, such as the SR protein SRSF3 [68,69].

Regarding members of the hnRNP family, altered expression of several hnRNPs has been found to favor carcinogenesis in several organs, including the liver. Thus, tumor suppressor hnRNPE1 (PCBP1) is down-regulated in several cancers. In contrast, oncogenes hnRNPE2 (PCBP2) and hnRNPK are up-regulated and promote tumorigenesis [70,71,72,73]. Nevertheless, in HCC, controversial results have been reported in this respect. Thus, both up- and down-regulation of hnRNPE2 in HCC have been reported by different studies [43,74]. Low expression of hnRNPK, hnRNPL, and hnRNPE2 has been associated with shorter OS of HCC patients [43]. The study of the role of hnRNPK in HCC development revealed that, after SUMOylation, hnRNPK could bind to p53 in HCC cells. This interaction ultimately leads to the accumulation and transactivation of p53 and hence inhibition of HCC cell proliferation [75]. Besides, miR-1249-3p could induce HCC cell proliferation and invasion through hnRNPK down-regulation [76]. These data are consistent with the positive impact of enhanced hnRNPK expression on HCC patient survival [43]. The role of hnRNPK in iCCA has also been described. O-glycosyl-N-acetylation of that protein has been associated with shorter survival of iCCA patients and increased iCCA progression and metastasis. The reason is that this post-translational modification promotes hnRNPK nuclear translocation, which acting as a transcription factor, induces the expression of several proteins involved in cell proliferation, migration, and apoptosis inhibition. Based on these findings, hnRNPK O-GlcNAcylation has been suggested as a druggable target to repress iCCA progression [44].

Enhanced expression of hnRNPAB in HCC has been associated with phenotypic epithelial-mesenchymal transition (EMT) in a SNAIL-dependent manner, leading to higher migration capacity and worse outcomes [77]. Furthermore, other members of this family of splicing factors, the polypyrimidine tract binding proteins (PTBP) are involved in HCC progression and metastasis. Thus, PTBP3 has increased expression in HCC, which is correlated with tumor size, metastasis, and lower survival [78]. Moreover, elevated levels of MTR4 promote HCC tumorigenesis through recruitment of PTPBP1 to its target pre-mRNA, which results in a metabolic switch in cancer precursor cells [79].

Among SR proteins, SRSF1, SRSF3, SRSF5, and SRSF9 are considered indirect oncogenes because they promote carcinogenesis through the enhanced generation of more oncogenic SVs of direct oncogenes, and their expression is elevated in several cancers [80,81,82,83,84]. Nevertheless, this does not seem to be the case in HCC, where a general down-regulation of these SR proteins has been found [69]. This is crucial because SRSF3 has been identified as an essential player for hepatocyte differentiation and metabolic function [85]. Thus, the aberrant splicing isoform of SRSF3, including exon 4 SRSF3-Iso2, which participates in the induction of genome instability, is up-regulated not only in HCC but already in the cirrhotic liver [68]. In mice, the loss of *Srsf3* induces AS of genes related to EMT and drives spontaneous HCC with aging [86]. A recent study has found a decreased SRSF3 activity in HCC cells due to an overexpression of PPM1G, a serine/threonine phosphatase involved in the dephosphorylation of splicing factors. As a result, cell proliferation, invasion, and metastasis of HCC cells were promoted through changes in the AS pattern of genes related to cell cycle control and transcriptional regulation [87]. Furthermore, SRSF2 expression levels have been shown to have prognostic value because SRSF2 up-regulation was inversely associated with patient survival [37,43]. Consistently, SRSF2 silencing using shRNAs decreased the tumorigenic potential of HCC cell lines in vivo by controlling the AS of target genes in a specific manner [37]. Altogether, these data indicate that SRSF2 protein could act as a cancer driver in human HCC. This seems to be a species-specific characteristic, as the loss of *Srsf2* has the opposite effect in mice, triggering hepatic progenitor cell activation and HCC onset [88].

**Table 1:** Impact of altered expression of spliceosome components and associated regulating proteins on the outcome of patients with liver cancer.

Gene	Change	Consequences	Clinical Impact	Cancer	Refs.
*ESRP1*	Up	Impaired exon inclusion	Enhanced migration; Distant metastasis	HCC iCCA	[89,90]
*hnRNPAB*	Up	Enhanced migration Enhanced EMT	Promote tumor recurrence	HCC	[77]
*hnRNPE2*	Controversial	Controversial	Change in OS	HCC	[43,74]
*hnRNPK*	Down	Transactivation of p53 Decreased proliferation	Shorter OS	HCC	[43]
*hnRNPK*	O-glycosyl-N-acetylation	Progression and Metastasis	Shorter OS	iCCA	[44]
*hnRNPL*	Down	Unknown	Shorter OS	HCC	[43]
*hnRNPM*	Up	Enhanced exon skipping	Enhanced migration; Distant metastasis	HCCi CCA	[91,92]
*PTBP3*	Up	Correlated with tumor size and metastasis	Shorter OS	HCC	[78]
*SF3B1*	Up	Enhanced proliferation, migration, and tumor growth	Shorter OS; Higher aggressiveness	HCC	[47]
*SF3B4*	Up	Promote tumorigenesis and metastasis	Reduced OS	HCC	[59,60]
*SLU7*	Down	Enhanced genome instability and de-differentiation	May favor hepatocarcinogenesis from cirrhotic liver	HCC	[66,67,68,69]
*SNRPB*	Up	Enhanced proliferation and migration	Shorter OS; Metastasis	HCC	[43]
*SNRPD1*	Up	Unknown	Shorter OS	HCC	[43]
*SRSF2*	Down	Enhanced proliferation Liver tumorigenesis in vivo	Longer OS	HCC	[43,88]
*SRSF3*	Down	Enhanced EMT and cell proliferation	Metastasis	HCC	[87]

Changes in gene expression were based on mRNA abundance. Down, down-regulation; EMT, Epithelial-mesenchymal transition; HCC, hepatocellular carcinoma; iCCA, intrahepatic cholangiocarcinoma; OS, overall survival; Up, up-regulation.

## 4. Altered Splicing of Genes Involved in Liver Carcinogenesis and Metastasis

Besides alterations in the splicing machinery, mutations in SS and *cis*-regulatory elements of target genes can also trigger cancer onset [93]. Thus, mutations (frequently at 3′SS) in several tumor suppressor genes, such as *TP53*, *ARID1A*, *PTEN*, *BRCA1*, *BRCA2*, *CDH1*, *MLH1*, and *FLCN*, are of particular relevance in carcinogenesis [94,95,96,97]. These mutations can affect the integrity of the SS, resulting in aberrant mRNA maturation [94,95,96,97] and subsequent impairment of their tumor suppression function, hence favoring cancer development [98]. Moreover, the cell cycle is highly dependent on pre-mRNA splicing and accurate AS. Several key cell cycle factors and their known SVs have been related to uncontrolled cell proliferation. Besides changes in their expression levels and the presence of somatic mutations, aberrant post-translational modifications, for instance, affecting chromatin dynamics, can also alter proper cell cycle control during carcinogenesis. Two primary connections link pre-mRNA splicing, cell cycle progression, and carcinogenesis. One is derived from the need for particular pre-mRNA splicing to allow the production of essential factors of the cell cycle that harbor introns. The second is AS of those critical factors required to produce the specific isoform needed for cell cycle progression (for a complete review, see [99]). Moreover, the proper function of the spliceosome component SF3B1, whose impact on carcinogenesis has been mentioned above, relies on its phosphorylated state at several sites, which varies during the cell cycle [100]. SF3B1 phosphorylation peaks at the G2/M phase in a CDK1-dependent manner and then decreases owing to PP2A and PP1 phosphatase activities as the cell cycle progresses through the G1/S phase [99].

Metastasis of HCC and iCCA cells has been related to AS events, mainly due to the appearance of SVs that promote EMT (Table 2 and Table 3). Alterations in the expression of different splicing factors, such as ESRP1 and hnRNPM, are critical in EMT activation. Mesenchymal phenotype is favored by hnRNPM due to its effect on enhancing exon skipping and TGFβ signaling, whereas ESRP1 favors epithelial phenotype by enhancing exon inclusion [90] (Table 1). Furthermore, ESRP1 can also act as an oncogene when it is overexpressed [89] (Table 1).

### 4.1. Role of SVs in the Onset, Progression, and Metastasis of HCC

#### 4.1.1. TP53

HCC development has been associated with aberrant splicing events affecting crucial genes (Table 2). For instance, the essential tumor suppressor gene *TP53* is frequently mutated in many types of cancers [101], including HCC [102]; several of its mutations affect SS located between introns 3 and 9, causing skipping of adjacent exons [101,103,104]. On the other hand, the *TP53* gene potentially encodes at least 12 p53 naturally occurring isoforms, resulting from the combination of four different N-terminal p53 forms (full-length, Δ40, Δ133, and Δ160) and three different C-terminal domains (α, β, and γ) [105]. The SV of *TP53* Δ40p53α, which lacks the 39 N-terminal amino acids corresponding to the first transactivation domain (TAD-I) of FL-p53, can suppress the proliferation of HCC cells [106].

#### 4.1.2. TP73

Two SVs of p73, ΔEx2p73, and ΔEx2/3p73, are highly expressed in HCC but are not found in healthy hepatocytes. Both isoforms are truncated proteins lacking the transactivation domain [66,107,108]. Their up-regulation in HCC accounts for the inhibition of p73 tumor-suppressive activity. Overexpression of human ΔEx2/3p73 in hepatocytes of transgenic mice results in the spontaneous development of HCC, supporting the oncogenic impact of these SVs [108]. Previous studies have unraveled the mechanism implicated in the up-regulation of these oncogenic isoforms and demonstrated that AREG induction in response to stress or inflammatory signals during liver damage is responsible for the down-regulation of SLU7, which in turn results in aberrant exon 2 skipping and hence the expression of the oncogenic isoform ΔEx2p73 [66].

#### 4.1.3. CDH17

Somatic mutations in critical sequences of oncogenes leading to their aberrant splicing also drive HCC development. Thus, the intestinal cell-cell adhesion protein, cadherin 17 (*CDH17*), is expressed in HCC as an SV lacking exon 7 (ΔEX7CDH17), which correlates with decreased OS and higher tumor recurrence [109]. The appearance of ΔEX7CDH17 has been associated with the presence in *CDH17* pre-mRNA of two single nucleotide polymorphisms (SNPs) located in exon 6 (rs 3214050) and the BPS of intron 6 (rs 2514813) [109]. The generation of this SV is considered among the genetic events contributing to the high prevalence of HCC in Asia [110].

#### 4.1.4. KLF6

As mentioned above, aberrant AS of genes regulating cell cycle arrest can also promote HCC development. Thus, the Krüppel-like factor 6 (KLF6) is a zinc-finger transcription factor that inhibits cell proliferation in part by transcriptional activation of p21, a cell cycle checkpoint protein. KLF6 function is abrogated in human cancers owing to increased AS that yields a dominant-negative isoform, KLF6 SV1, which antagonizes full-length KLF6-mediated growth suppression [111], presumably by promoting KLF6 degradation [112]. The ratio between SV1 and wild-type KLF6 was increased in HCV-associated HCC, which was related to higher tumor aggressiveness. Remarkably, elevated ratio SV1 and wild-type KLF6 promoted liver tumorigenesis in mice [112]. In contrast, the SV2 KLF6 variant is down-regulated in human HCC samples compared to adjacent non-tumor liver tissue. It has anti-tumorigenic activity, inducing cell cycle arrest by p21 activation and increasing pro-apoptotic factors, such as BAX [113].

#### 4.1.5. FGFR2

The tyrosine kinase receptor fibroblast growth factor receptor 2 (FGFR2) undergoes AS generating two SVs (FGFR2-IIIb and FGFR2-IIIc) by incorporating mutually exclusive exons. EMT is associated with AS of FGFR2 switching from the epithelial- (FGFR2-IIIb) to the mesenchymal-type (FGFR2-IIIc) SV, which further promotes invasion and metastasis [114]. Although several proteins can affect AS of FGFR2, ESRP1 is the primary regulator, which promotes FGFR2-IIIb expression and hence favors epithelial phenotype [115,116]. In HCC, FGFR2-IIIb down-regulation has been associated with higher vascular invasion and more advanced tumor stage. Moreover, FGFR2-IIIb re-expression in HCC cells in vitro reduced proliferation and migratory potential [117].

#### 4.1.6. FGFR3

Another tyrosine kinase receptor abnormally up-regulated in HCC is the fibroblast growth factor receptor 3 (FGFR3) [118], which also undergoes AS, plays an important role in cell proliferation, differentiation, and angiogenesis. The IgG-like-III domain of FGFR3, which is required for binding to its ligands (FGFs), is encoded by two separate exons, i.e., exon 8 and exon 9. The alternative selection of one of these exons results in FGFR3-IIIb and FGFR3-IIIc SVs, respectively. The expression levels of these SVs, both separately or in combination, are higher in 53% of all HCCs than in adjacent non-tumor liver tissue, especially in cases with early tumor infiltration, metastasis, and recurrence at the time of surgery [118]. Interestingly, FGFR3-IIIb overexpression in HCC cell lines using lentiviral constructs leads to enhanced proliferation. Moreover, both SVs could inhibit apoptosis and improve the tumorigenic activity of these HCC cells in vivo. Besides, blockade of FGFR3-IIIb/c dramatically reduced tumor growth [118], which suggests that these SVs act as oncogenes in HCC and are potential therapeutic targets. Moreover, aberrant AS of FGFR3 could also lead to the mutant FGFR3Δ7–9 variant, which has carcinogenic activity and has been associated with enhanced invasiveness in vitro and metastasis in vivo [119]. This isoform links exon 6 to exon 10 directly, resulting in a complete deletion of the IG-like-III domain. Nevertheless, the FGFR3Δ7–9 variant can be self-activated, independently of the FGF ligand binding. FGFR3Δ7–9 activates the AKT pathway and indirectly decreases the expression of the tumor suppressor PTEN [120]. In this way, FGFR3Δ7–9 enhances HCC cell proliferation and tumor growth in vivo [119]. Moreover, FGFR3Δ7–9 expression is accompanied by lower E-cadherin levels and a higher expression of stem/mesenchymal markers such as SNAIL and MMP-9 [119].

#### 4.1.7. DNMT3b3

Some HCC-associated SVs have already been detected in preneoplastic stages, suggesting their possible early contribution to liver malignancy. For example, up-regulation of DNA methyltransferase 3b3 (DNMT3b3) has been described in the liver of patients with hepatitis and chronic cirrhosis, as well as in HCC. This was partly due to an elevated expression of DNMT3b4, a splice variant of DNMT3b lacking conserved methyltransferase motifs IX and X, which has been correlated with the degree of DNA hypomethylation in pericentromeric satellite regions both under precancerous conditions and in HCC [121].

#### 4.1.8. PDSS2

Another example concerns the AS of prenyl diphosphate synthase subunit 2 (PDSS2), an essential enzyme in coenzyme Q10 synthesis involved in hepatocarcinogenesis [122]. PDSS2 down-regulation has been associated with a poor prognosis in HCC. Interestingly, an SV of PDSS2 lacking exon 2 (PDSS2Δ2) has been detected in HCC, and its overexpression was associated with shorter OS [123]. Apart from its pro-carcinogenic activity [122], PDSS2∆2 favors cell migration in vitro and metastasis in vivo, presumably, through Wnt/β-catenin and NF-Kβ signaling pathway [123].

#### 4.1.9. AXL

The AXL tyrosine kinase receptor has attracted much attention as a potential therapeutical target. Skipping of exon 10 in *AXL* pre-mRNA gives rise to the AXL short (AXL-S) SV, which is up-regulated in HCC cells by hnRNPI (PTBP1) resulting in the promotion of cell migration [124]. Consistently, AXL-S silencing in HCC cells significantly inhibited pulmonary metastasis in a xenografted nude mouse model [124].

#### 4.1.10. NF2

The tumor suppressor moesin-ezrin-radixin-like protein (Merlin) encoded by the *NF2* gene is down-regulated in HCC [125]. Interestingly, an SV lacking exons 2, 3, and 4 (MerlinΔ2–4), identified in HCC, has the opposite effect on liver tumor progression to that of the wild-type isoform [125]. Thus, knocking-down Merlin or overexpressing MerlinΔ2–4 promotes cell migration and invasion through Twist1 up-regulation. Furthermore, MerlinΔ2–4 also stimulates the activation of β-catenin and the expression of stemness-related genes. Remarkably, using a pulmonary metastatic mouse model, the expression of Merlin reduced metastasis, whereas that of MerlinΔ2–4 induced distant metastasis [125].

#### 4.1.11. XBP1

The SV of the X-box binding protein 1 (XBP1s) is a well-known activator of UPR (unfolded protein response), an adaptive response and defense mechanism through which tumor cells can survive under adverse conditions of enhanced ER stress. XBP1s expression is markedly high in HCC, where its up-regulation correlates with the existence of distant metastasis and poor prognosis. This could be due to the ability of XBP1 to induce EMT and migration in HCC cells [126].

#### 4.1.12. OPN

Three SVs of osteopontin (OPN), a glycoprotein of the extracellular matrix involved in proliferation, angiogenesis, and invasion, have been described. OPN-a contains all exons, whereas OPN-b and OPN-c lack exons 5 and 4, respectively. Overexpression of OPN-a and OPN-b has been associated with greater invasiveness in HCC [127].

#### 4.1.13. CD44

CD44 is a membrane receptor glycoprotein considered a cancer-stem cell (CSC) marker that binds to proteins and other ligands to activate signal transduction the control essential functions in proliferation, migration, and tumor invasion [128]. AS of CD44 can generate up to twelve SVs with different biological activities [129]. The switch between variants has been associated with EMT regulation [130]. In HCC, CD44v3 promotes metastasis, and its up-regulation is favored by the overexpression of nicotinamide N-methyltransferase (NNMT) [131]. Interestingly, studies in non-liver tumor tissue suggest that the generation of different CD44 variants may be affected by overexpression of ESRP1 and hnRNPM, two critical splicing factors involved in EMT regulation [132,133,134].

#### 4.1.14. Genome Instability

Moreover, isoforms derived from aberrant splicing can also promote genome instability in HCC [135]. This is the case of the HCC-induced isoform SRSF3-Iso2, which has been implicated in the incorporation of introns 1 and 2 into *CDCA5* mRNA, resulting in reduced levels of sororin, a protein essential to maintain sister chromatid cohesion [68].

Gene transcription and alterations in the expression of RBPs are important sources of replication stress and genome instability related to the pathogenesis of aging and neoplastic diseases, constituting one of the hallmarks of cancer [68]. Moreover, isoforms derived from aberrant splicing can also promote genome instability in HCC [135]. In this context, the splicing regulator SLU7 safeguards genomic integrity, preventing the formation of R-loops and the induction of DNA damage and ensuring proper chromosome segregation during mitosis [68]. Its expression is significantly down-regulated in HCC [66], which results in enhanced oxidative stress, DNA damage, mitotic aberrations, and genome instability, leading to an enhanced risk of carcinogenesis. Moreover, SLU7 is essential to prevent an increased expression of SRSF3 truncated proteins by dysregulation of SRSF3 splicing. The HCC-induced isoform SRSF3-Iso2 is involved in the incorporation of introns 1 and 2 into *CDCA5* mRNA resulting in reduced levels of sororin, a protein essential to maintain sister chromatid cohesion [68]. The preservation of normal hepatic SRSF3 function appears to be critical to prevent liver carcinogenesis, since specific deletion of this gene in hepatocytes results in the spontaneous development of liver injury and HCC [86].

Other AS events related to chromosome instability occurring in HCC include exon 4 deletion (MAD1beta) in the mitotic arrest deficient 1 (*MAD1*) gene [136] and exon 6 deletion (AURKB-Sv2) in the serine/threonine kinase Aurora B (AURKB) [137].

**Table 2:** Alternative splicing of genes involved in hepatocellular carcinoma development and metastasis.

Gene	Variant	Splicing Event	Consequences	Refs.
*AXL*	AXL-S	Exon 10 skipping	Enhanced migration; Distant metastasis	[124]
*CD44*	CD44v3	Lacks multiple coding exons compared to variant 1	Promotes metastasis	[131]
*CDH17*	ΔEX7CDH17	Exon 7 skipping	Decreased OS; High tumor recurrence	[109]
*DNMT3b3*	DNMT3b4	Loss of methyltransferase motifs IX and X	Induced DNA hypomethylation and carcinogenesis	[121]
*FGFR2*	FGFR2-IIIb/IIIc	Mutually exclusive exons	Promoted invasion and metastasis	[114]
*FGFR3*	FGFR3-IIIb	Alternative exon 8 inclusion	Increased proliferation in vitro; Increased tumor growth in vivo; Apoptosis inhibition	[118]
*FGFR3*	FGFR3-IIIc	Alternative exon 9 inclusion	Increased tumor growth in vivo; Apoptosis inhibition	[118]
*FGFR3*	FGFR3Δ7–9	Exons 7 to 9 skipping	Activation of AKT and decreased expression of PTEN; Enhanced cell proliferation and tumor growth; Enhanced cell motility; Activation of EMT; Distant metastasis in vivo	[119]
*KLF6*	KLF6-SV(1,2)	Exons 2 (partial) and 3 skipping	Enhanced tumorigenesis and aggressiveness	[112,113]
*NF2*	Merlin Δ2–4	Exons 2–4 skipping	Enhanced migration and invasion; Activation of stemness; Distant metastasis.	[125]
*OPN*	OPN-b	Exon 5 skipping	Enhanced invasiveness	[127]
*PDSS2*	PDSS2Δ2	Exon 2 skipping	Enhanced migration; Distant metastasis; Decreased OS	[123]
*TP53*	Short SVs Δ40p53α	Several exons skipping	Various effects on cell proliferation	[101,103,104,106]
*TP73*	ΔEx2/3p73	Exon 2 and 3 skipping	Tumor development in vivo	[108]
*XPB1*	XBP1s	Intron skipping and frameshift	Distant metastasis; Poor prognosis	[112]

EMT, epithelial-mesenchymal transition; OS, overall survival; SV, splicing variant.

### 4.2. Role of SVs in the Onset, Progression, and Metastasis of iCCA

Although more than six hundred genes have been reported to undergo AS in iCCA [38], there is scarce information on their role in iCCA initiation and metastasis. Several SVs associated with iCCA development are commented below and summarized in Table 3.

#### 4.2.1. FOXP3

Recently, a prognosis signature of genomic instability-related AS events has been identified for CCA data available at TCGA, without discriminating the anatomical location of the tumor [7]. Moreover, this signature could be associated with the presence of infiltrating immune cells and the expression of PD-L1 [7], having, therefore therapeutic impact. Thus, skipping exons 3 and 4 in *FOXP3* mRNA leads to a frameshift in the open reading frame (ORF) and hence in the amino acid sequence of FOXP3 results in a different protein involved in the development of CD25+ regulatory T cells [138]. This SV has also been detected in CCA cell lines [139]. Although the implication of this SV in iCCA onset is not well known, in other tumors, such as melanoma, this variant has been associated with immune suppression [138].

#### 4.2.2. TFF2

Despite aberrant splicing is usually related to cancer promotion, some cases show the opposite association. For instance, this is the case of Trefoil factor 2 (TFF2), a secreted protein of the gastrointestinal mucosa that is up-regulated in many cancer types, including iCCA [140]. The SV generated by exon 2-skipping (ΔEX2TFF2) of *TFF2* pre-mRNA, which results in the formation of a premature stop codon, has been identified in iCCA [141]. This non-functional TFF2 SV, in which most of the protein structure is lost, cannot mediate cell proliferation like the canonical wtTFF2 isoform does, which results in tumor progression inhibition. Interestingly, ΔEX2TFF2 expression correlated with more prolonged survival of iCCA patients. In contrast, the abundance of wtTTF2 in tumor tissue was associated with lower OS of these patients, suggesting that ΔEX2TFF2 could counteract the cancer-promoting activity of wtTFF2 in iCCA [141].

#### 4.2.3. BAP1

Germline mutations in the tumor suppressor gene *BAP1,* encoding the BRCA1 associated protein-1 (BAP1), cause the so-called BAP1 tumor predisposition syndrome (BAP1-TPDS), characterized by an increased propensity to develop several types of cancer. In a human mesothelioma cell line, mutations in *BAP1* pre-mRNA have been shown to contribute to tumorigenesis by disrupting normal splicing [142]. The mutations found in BAP1-TPDS patients affect ubiquitin carboxyl hydrolase (UCH) and nuclear localization signal (NLS) domains and create a non-functional truncated protein, disrupting BAP1 deubiquitinating activity and nuclear localization, both required for BAP1 tumor suppressor function [143]. Evidence for the relationship between germline mutations in 3′SS or 5′SS of exon 4 of *BAP1* pre-mRNA and the development of iCCA has been reported [144].

#### 4.2.4. AGR2

Regarding metastasis, several connections between AS and iCCA have been reported. Anterior gradient protein 2 (AGR2) is a member of the protein disulfide isomerases family, which is involved in protein modification and folding. AGR2 is up-regulated in a wide variety of solid tumors, where it is involved in their progression and metastasis [145]. AGR2 has several exon-skipping SVs, from AGR2vA to AGR2vH. The SV lacking exons 1 and 4–7 (AGR2vH) has been found in tumor samples derived from iCCA patients [146]. When overexpressed in CCA cell lines, this SV promotes not only survival and apoptosis evasion, thereby favoring cancer progression, but also metastasis-associated phenotypes [147], by increasing cell motility and up-regulating the expression of the mesenchymal marker vimentin [148]. On the contrary, down-regulation of AGR2vH has been found to reduce cell migration capacity [148].

#### 4.2.5. WISP1

Wnt-inducible secreted protein 1 (WISP1, also known as CNN4) belongs to the cysteine-rich CCN family of proteins, which are involved in cell-matrix interaction and mediate cell adhesion, migration, proliferation, and apoptosis [149]. The SV WISP1v, lacking exon 3, has been found in approximately 50% of iCCA samples examined, whereas it was absent in the adjacent non-tumor liver tissue [150]. In the same study, *WISP1v* mRNA abundance was associated with perineural and lymphatic invasion and reduced OS after surgery. Moreover, WISP1v expression in HuCCT1 cells induced rapid growth and migration through p38 MAPK signaling activation [150].

#### 4.2.6. PKM

PKM2 is an SV of pyruvate kinase (PKM), a rate-limiting enzyme in glycolysis. Immunohistochemical analysis of iCCA and hilar CCA revealed that PKM2 was up-regulated in tumor tissue compared with adjacent non-tumor liver tissue [151,152]. Higher PKM2 has been associated with enhanced proliferation and invasiveness of CCA cells, whereas patients presented shorter OS [151,152], which was related to lymph node invasion and distant metastasis [151]. Consistently, wound healing experiments showed that CCA cells transfected with shRNAs against PKM2 had lower motility than those with endogenous enzyme levels. Finally, in an orthotopic xenografted-mouse model of CCA, silencing of PKM2 expression remarkably inhibited both tumor growth and metastasis [152].

#### 4.2.7. PTGER3

The AS of pre-mRNA of prostaglandin E2 receptor 3 (EP3, *PTGER3*) can generate eleven SVs. The SV EP3–4, which has been detected in several human tissues, such as gastric mucosa, mammary artery, and pulmonary vessels. This shorter mRNA includes exon 1, 2a, 5, and 10, which results in a truncated protein [153]. The overexpression of this SV in iCCA cells (CCLP1 and HuCCT1) enhances the expression of c-Myc and SNAIL, which supports the role of EP3–4 in iCCA progression and metastasis [154].

#### 4.2.8. CD44

Among CD44 SVs, CD44v6 promotes EMT and activates the TGF-β pathway. CD44v6 expression was specifically found in proliferating cholangiocytes and CCA samples but not in healthy bile ducts [155]. In addition, patients with iCCA expressing CD44v6 and CD44v8–10 had shorter relapse-free survival (RFS) and OS than those without expression of these CD44 SVs. Moreover, the levels CD44v6 and CD44v8–10 proteins in serum were significantly increased in the recurrence group for early-stage iCCA, suggesting that soluble forms of CD44v6 and CD44v8–10 could serve as markers of post-operative iCCA recurrence [156].

**Table 3:** Alternative splicing of genes involved in intrahepatic cholangiocarcinoma development and metastasis.

Gene	Variant	Splicing Event	Consequence	Refs.
*AGR2*	AGR2vA to H	Several combinations of exons 2 to 7 skipping	Affect cancer cell survival and migration	[148]
*BAP1*	BAP1 p.E685V	Multiple SV lacking exons 14–17	Promote tumorigenesis	[142]
*CD44*	CD44v6; CD44v8–10	Exon 6 skipping; exon 8–10 skipping	Decreased OS and RFS; Tumor recurrence	[156]
*FOXP3*	FOXP3	Exons 2–4 skipping	Immune suppression	[138,139]
*PKM*	PKM2	Mutual exclusive exons: exon 9 skipping/exon 10 retention	Decreased OS; Enhanced risk of metastasis	[151,152]
*PTGER3*	EP3–4	Contains exon 1, 2a, 5, and 10	Enhanced cell proliferation, migration, and invasion	[153,154]
*TFF2*	ΔEX2TFF2	Exon 2 skipping	Increased OS	[141]
*WISP1*	WISP1v	Exon 3 skipping	Decreased OS; Induction of invasion	[150]

OS, overall survival; RFS, relapse-free survival; SV, splicing variant.

## 5. Altered Splicing of Genes Involved in the Resistance of Liver Cancer to Anticancer Drugs

The lack of response of liver cancer to drug treatment is one of the fundamental reasons for the high mortality caused by these tumors when they are diagnosed in an advanced non-operable stage, which unfortunately is a frequent situation in everyday clinical practice nowadays. The impact of AS on drug resistance mechanisms of cancer cells has recently been reviewed [157]. Regarding HCC and iCCA, the available data are still scarce. Hindering the access of anticancer drugs to their intracellular targets notably reduces their efficacy. This mechanism of pharmacoresistance (MPR), classified as MPR-1a [158,159], plays a crucial role in the lack of response. The uptake by HCC cells of sorafenib, a first-line drug against HCC, is mainly carried out through the organic cation transporter 1 (OCT1, *SLC22A1*) [160]. AS has a marked impact on this process because several OCT1 SVs are generated, most of them by exon skipping (exon 10, exon 9, or exon 9 + 10 skipping) and are translated into shorter OCT1 isoforms without transport activity. These non-functional SVs are abundantly expressed in HCC and iCCA [161]. Apart from sorafenib, a recent study has demonstrated that the failure of therapies targeting the androgen receptor (AR) in HCC could be associated with the expression of truncated AR-SVs [162] capable of driving AR signaling in the presence of antiandrogen therapy.

Drug resistance has also been associated with metabolic reprogramming in HCC, and more specifically, with the switch from oxidative phosphorylation to aerobic glycolysis [163,164]. As previously mentioned, PKM2 is a rate-limiting enzyme in the glycolytic pathway. This PKM isoform is produced by an AS mechanism that can be triggered, for instance, by down-regulation of the splicing factor SLU7 [69] or up-regulation of the RNA helicase MTR4 both events described in HCC [79].

Immune checkpoint inhibitors (ICI)-based immunotherapy is emerging as a promising strategy for treating advanced liver tumors [165,166]. A recent study comprehensively evaluated the differential AS events between HCC or iCCA tissues and the corresponding normal tissues focusing on immune features [167]. Interestingly, this work identified AS-related events that were not only associated with the prognosis of these patients but also with the formation of the differential immune microenvironment of these tumors. Unraveling the involvement of AS alterations in shaping the immune landscape of liver cancers may contribute to understanding their pathogenesis and improving the efficacy of ICI-based therapies.

## 6. Conclusions and Perspectives

The variability present in our proteome, thanks to the intervention of the splicing machinery during pre-mRNA maturation, constitutes a notable physiological advantage conferring the required flexibility to many cellular functions. Nevertheless, on the other side of the coin, AS may also provide tumor cells with dynamic and adaptative phenotypic traits that permit them to escape the host’s immune system and resist pharmacological treatment. The expression of SVs can lead to the loss of cell viability but also the development of a more malignant phenotype, owing to enhanced proliferative activity, reduced activation of pro-apoptotic pathways, enhanced capacity for local invasion, long-distance metastasis, and drug resistance. Several hundred splicing events have been described to occur differentially in liver cancer compared to normal liver tissue. Some of them have also been detected in other cancers. However, the complexity of the pre-mRNA maturation process, the differences in the transcriptome among tissues, and the heterogeneity concerning each type of cancer prevent us from reaching any general conclusion.

The study of the impact of AS on protein domain boundaries could offer the rationale to interpret the relevance of SV in liver cancer biology. Although, as commented above, there is some available information in this respect, for instance, regarding FGFR3 [119,120] and BAP1 [143] variants, this analysis requires individual gene investigation, which in many cases is still missing.

Identifying the impact of AS on cancer cell biology is crucial for the development of novel strategies targeting these mechanisms. Interfering with pro-carcinogenic AS events could reduce the malignancy of HCC and iCCA and eventually improve the outcomes of these patients. In this respect, there are already ongoing attempts to develop small molecules able to interact with splicing factors to modulate AS. However, there is still a long way for these drugs to reach clinical practice because they lack cancer cell specificity, and most of their molecular targets are constitutive elements of the spliceosome, whose pharmacological manipulation can have an impact on the correct expression of many different genes resulting in noxious effects for healthy cells [168,169].

## Abbreviations

3′SS, 3′ acceptor site; 5′SS, 5′ donor site; AR, androgen receptor; AREG, amphiregulin; AS, alternative splicing; AURKB, aurora kinase B; AXL, AXL tyrosine kinase receptor; AXL-S, AXL short; BAP1, BRCA1 associated protein-1; BAP1-TPDS, BAP1 tumor predisposition syndrome; BPS, branch point site; CCA, cholangiocarcinoma; CDH17, cadherin 17; CES, Carboxylesterase; CSC, cancer stem cell; DNMT, methyltransferase; EGFR, epidermal growth factor receptor; EMT, epithelial-mesenchymal transition; ENT1, Equilibrative nucleoside transporter 1; ER, estrogen receptor; ESE, exonic splicing enhancers; ESS, exonic splicing silencers; FGFR2, fibroblast growth factor receptor 2; HCC, hepatocellular carcinoma; HCCDB, Integrative Molecular Database of HCC; hnRNP, heterogeneous nuclear ribonucleoprotein; HR, homologous recombination; iCCA, intrahepatic cholangiocarcinoma; ICI, immune checkpoint inhibitors; ISE, intronic splicing enhancers; ISS, intronic splicing silencers; KLF6, Krüppel-like factor 6; lncRNA, long noncoding RNA; Merlin, moesin-ezrin-radixin-like protein; MPRs, mechanisms of pharmacoresistance; NLS, nuclear localization signal; NNMT, nicotinamide NN-methyltransferase; NSCLC, non-small cell lung cancer; OCT1, Organic cation transporter 1; OPN, osteopontin; ORF, open reading frame; OS, overall survival; PARPi, PARP inhibitors; PDSS2, prenyl diphosphate synthase subunit 2; PKM, pyruvate kinase gene; PPT, polypyrimidine tract; PRPF, pre-mRNA processing factors; PTBP, polypyrimidine tract binding proteins; PTGER3, Prostaglandin E2 receptor EP3; RBP, RNA-binding protein; RFS, relapse-free survival; Sf1, splicing factor 1; SLU7, step II splicing regulator SLU7; SNP, single nucleotide polymorphism; snRNP, small nuclear ribonucleoprotein; snRNA, small nuclear RNA; SV, splicing variant; TFF2, Trefoil factor 2; TKIs, tyrosine kinase inhibitors; TP, Thymidine; UCH, ubiquitin carboxyl hydrolase; UPR, unfolded protein response; WISP1, Wnt-inducible secreted protein 1; XBP1s, X-box binding protein 1.
